# Subjective Outcome Evaluation of the Project P.A.T.H.S.: Findings Based on the Perspective of the Program Implementers (Secondary 1 Program)

**DOI:** 10.1100/tsw.2010.22

**Published:** 2010-02-12

**Authors:** Sandra K.M. Tsang, Eadaoin K.P. Hui, Daniel T.L. Shek, Bella C.M. Law

**Affiliations:** ^1^ Department of Social Work and Social Administration, Faculty of Social Sciences, University of Hong Kong, Shanghai, China; ^2^ Faculty of Education, University of Hong Kong, Shanghai, China; ^3^ Department of Applied Social Sciences, Hong Kong Polytechnic University, Shanghai, China; ^4^ Department of Sociology, East China Normal University, Shanghai, China; ^5^ Kiang Wu Nursing College of Macau, Macao

**Keywords:** adolescents, positive adolescent training, program evaluation, program implementers, student development, subjective outcome evaluation

## Abstract

The aim of the current study was to replicate the subjective outcome evaluation based on program implementers in the first year (2006/07 school year) of the Full Implementation Phase (Secondary 1 level) of the Project P.A.T.H.S. (Positive Adolescent Training through Holistic Social Programmes). After the completion of the Tier 1 program in the 2007/08 school year, 1324 implementers from 213 schools completed a Subjective Outcome Evaluation Form for instructors in order to assess their views of the program, themselves, and the perceived effectiveness of the program. Reliability test indicated the questionnaire was internally consistent. The results showed that, similar to the first year of implementation, high proportions of the respondents had positive perceptions of the program and their own performance. Regarding the perceived effectiveness of the program, roughly 90% of the respondents thought the program was helpful. A statistically significant increase in positive responses was also found in some items of perceived effectiveness in the second year of implementation. Possible factors contributing to such changes, including accumulation of experience and skill enhancement of the implementers, as well as stronger support from the schools, are discussed.

## INTRODUCTION

"P.A.T.H.S. to Adulthood: A Jockey Club Youth Enhancement Scheme" is a pioneering research-based positive youth development (PYD) program in a Chinese context. The word "P.A.T.H.S." stands for

"Positive Adolescent Training through Holistic Social Programmes". It is funded by The Hong Kong Jockey Club Charities Trust, aimed at promoting holistic development among adolescents in Hong Kong. The program consists of two tiers. The Tier 1 program, the focus of the current study, is a universal PYD program, normally with 20 h of training in each school year for students in Secondary 1 to 3 to participate. Tier 2 targets those students identified with greater psychosocial needs. There are two implementation phases: the Experimental Implementation Phase and the Full Implementation Phase. The Full Implementation Phase was executed in the 2006/07 school year, with 207 schools joining the Secondary 1 Tier 1 Program.

A subjective outcome evaluation based on the program implementers was done in order to assess the implementers' views of the program, themselves, and the perceived effectiveness of the program in the first year of implementation. According to Shek and Ma[1], the importance of examining the experiences of program implementers, the stakeholders of the program, especially in the context of human services is sixfold: (1) implementers have the first-hand experience regarding the implementation process of the program (p. 496); (2) because of the professionalism and experience of the program implementers, their views in certain aspects may be more accurate than the program participants; (3) the inclusion of implementers' comments can give them a sense of fairness and respect; (4) this kind of evaluation can help to provide a transparent and accurate picture on the implementation quality; (5) according to the principle of triangulation, collecting the views of the program implementers can help to increase the credibility of the evaluation, since the evaluation is done based on different data sources; and (6) the implementers' view towards the program would, consciously or unconsciously, affect how the program is launched. The evaluation may provide insight into the context in which the program operates (p. 493).

The pivotal role of implementers in program effectiveness is also demonstrated in a study by Hui[2]. She examined the introduction of developmental guidance as a formal curriculum into a Hong Kong secondary school and identified several factors that facilitated the program implementation. First, teachers' acceptance of the beliefs promoted in the program was necessary. Teachers were more involved in implementation if they identified with the belief. Second, teachers' knowledge and familiarity with the content and the teaching approach, and their active participation in the activities, were important in order to encourage students' involvement. Third, the principals' support and administrative arrangement to lessen teachers' workload and deal with teachers' concerns proactively were essential. The establishment of the "dual classroom teacher" system facilitated better communication, mutual support between classroom teachers and their engagement in teamwork, and alleviated their pressure in preparation and management. Finally, the school played an important role in offering experiences to strengthen students' learning and in cultivating a learning environment filled with positive interpersonal relationships. The program delivery should involve all students and all school personnel.

The above experience revealed that apart from the program content, the implementers and the school system also greatly affect students' learning and development. Referring to Bronfenbrenner's ecological model, the interactions between people and the various systems within their environment have tremendous impact on human behavior[3]. Within this perspective, people and their environment continually influence one another in a biodirectional, transactional, or mutual manner. The model also emphasizes the importance of an individual's life settings (i.e., microsystem), such as family and school, the interface between these settings (i.e., mesosystem), as well as the overarching institutional patterns of the culture or subculture of which the other systems are the concrete manifestation (i.e., macrosystem) in influencing behavior. Hence, in order to facilitate the positive growth of teenagers effectively, developmental programs of good quality, experienced and devoted implementers with relevant knowledge and understanding of the program, a strong and comprehensive system support from school, and participation of parents, the community, and society are of great importance.

In the subjective outcome evaluation of the first year of implementation of the Full Implementation Phase (Secondary 1 level), the findings based on program implementers from 207 schools are encouraging. In general, a high proportion of the implementers had positive perceptions towards the program and themselves as the program implementers, and roughly four-fifths of the implementers regarded the program as helpful to the program participants[1]. To get a clearer and broader picture on how the implementers view the program, themselves as the implementers, and the perceived effectiveness on program participants, a replication study was done by collecting subjective outcome evaluation data based on program implementers from 213 schools participating in the second year (2007/08 school year) of the Full Implementation Phase (Secondary 1 level). The implementers' responses and a comparison between the implementers' perception in the two implementation periods were the foci of the current study.

## METHOD

### Respondents and Procedures

There were 213 schools that joined the Secondary 1 Program of the Project P.A.T.H.S. in the second year of the Full Implementation Phase in the 2007/08 school year. The mean number of students per school was 171.05 (range: 16–267), with an average of 4.69 classes per school (range: 1–8). Among them, 105 schools adopted the full program (i.e., 20-h program involving 40 units), while 108 schools adopted the core program (i.e., 10-h program involving 20 units). The mean number of sessions used to implement the program was 23.61 (range: 5–60). While 116 (54.50%) schools incorporated the program into the formal curriculum (e.g., Liberal Studies, Life Education), 97 schools (45.50%) used other modes (e.g., using form teachers' periods and other combinations) to implement the program. A total of 1630 implementers carried out the program in the schools. The mean numbers of social workers and teachers implementing the program per school were 2.00 (range: 0–8) and 5.63 (range: 0–28), respectively.

After the Tier 1 Program was completed, the implementers were invited to respond to a subjective outcome evaluation questionnaire. A total of 1324 implementers responded to the Subjective Outcome Evaluation Form for instructors (Form B) developed by the research team. The data collection was normally carried out after the completion of the program. To facilitate the program evaluation, the research team developed an evaluation manual with standardized instructions for collecting the subjective outcome evaluation data[4]. In addition, adequate training was provided to the implementers during the 20-h training workshops on how to collect and analyze the data collected by the questionnaire.

### Instruments

The Subjective Outcome Evaluation Form for instructors, comprising 39 items on a Likert scale, was used in the present study to measure implementers' perception towards (a) the program, (b) their own performance as program implementers, (c) the effectiveness of the program on students, (d) the extent to which they would recommend the program to other students with similar needs, (e) the extent to which they would teach similar programs in future, and (f) the extent to which the program implementation has helped the implementers' professional growth[4].

## RESULTS

Results showed that the questionnaire was internally consistent. The Cronbach's alpha for the whole Form B was 0.98, with mean interitem correlation = 0.51. The findings are also consistent with the previous study (alpha = 0.97, mean interitem correlation = 0.47).

The quantitative findings based on the closed-ended questions are presented in this paper. There are several observations that can be highlighted from the findings. First, the respondents generally had positive perceptions of the program (Table 1), including clear objectives of the teaching units (94.63%), well-planned teaching activities (89.54%), and high peer interaction in the program (88.09%).

**TABLE 1**
**Summary of the Views of the Implementers towards the Program**

| | 1 | | 2 | | 3 | | 4 | | 5 | | 6 | | Participants with Positive Responses (Options 4–6) | |
|---|---|---|---|---|---|---|---|---|---|---|---|---|---|---|
| | Strongly Disagree | | Disagree | | Slightly Disagree | | Slightly Agree | | Agree | | Strongly Agree | | | |
| | n | % | n | % | n | % | n | % | n | % | n | % | n | % |
| The objectives of the curriculum are very clear (n = 1321) | 1 | 0.08 | 14 | 1.06 | 56 | 4.24 | 295 | 22.33 | 853 | 64.57 | 102 | 7.72 | 1250 | 94.63 |
| The design of the curriculum is very good (n = 1322) | 10 | 0.76 | 44 | 3.33 | 148 | 11.20 | 523 | 39.56 | 548 | 41.45 | 49 | 3.71 | 1120 | 84.72 |
| The activities were carefully planned (n = 1319) | 4 | 0.30 | 31 | 2.35 | 103 | 7.81 | 460 | 34.87 | 650 | 49.28 | 71 | 5.38 | 1181 | 89.54 |
| The classroom atmosphere was very pleasant (n = 1318) | 4 | 0.30 | 27 | 2.05 | 138 | 10.47 | 438 | 33.23 | 606 | 45.98 | 105 | 7.97 | 1149 | 87.18 |
| There was much peer interaction among the students (n = 1318) | 2 | 0.15 | 29 | 2.20 | 126 | 9.56 | 487 | 36.95 | 588 | 44.61 | 86 | 6.53 | 1161 | 88.09 |
| Students participated actively during lessons (including discussions, sharing, games, etc.).(n = 1307) | 5 | 0.38 | 41 | 3.14 | 140 | 10.71 | 461 | 35.27 | 567 | 43.38 | 93 | 7.12 | 1121 | 85.77 |
| The program has a strong and sound theoretical support (n = 1303) | 7 | 0.54 | 35 | 2.69 | 141 | 10.82 | 515 | 39.52 | 516 | 39.60 | 89 | 6.83 | 1120 | 85.96 |
| The teaching experience I encountered enhanced my interest in the course (n = 1302) | 22 | 1.69 | 61 | 4.69 | 179 | 13.75 | 504 | 38.71 | 476 | 36.56 | 60 | 4.61 | 1040 | 79.88 |
| Overall speaking, I have very positive evaluation of the program (n = 1310) | 15 | 1.15 | 73 | 5.57 | 181 | 13.82 | 516 | 39.39 | 483 | 36.87 | 42 | 3.21 | 1041 | 79.47 |
| On the whole, students like this curriculum very much (n = 1307) | 13 | 0.99 | 58 | 4.44 | 197 | 15.07 | 530 | 40.55 | 470 | 35.96 | 39 | 2.98 | 1039 | 79.50 |

Second, a high proportion of the respondents had a positive evaluation of their performance (Table 2). For example, 99% of the respondents felt ready to offer help to their students, 98.62% of the respondents expressed that they were concerned about the students, and 96.53% believed that they had very good professional attitudes.

Third, as shown in Table 3, many respondents perceived that the program promoted the development of students, including bonding (89.58%), social competence (92.80%), emotional competence (89.13%), moral competence (90.59%), self-understanding (92.47%), and overall development (93.53%).

When focusing on respondents' ratings about whether the program was "helpful" or "very helpful", at least one-third to one-half of the respondents perceived that the program promoted students' development, including social competence (52.26%), self-awareness (52.24%), self-determination (40.96%), cognitive competence (39.25%), self-confidence (35.86%), and overall development (48.78%). Independent sample t-test was administered in order to compare the rating of the respondents in the first year of implementation

**TABLE 2**
**Summary of the Views of the Implementers about Themselves**

| | 1 | | 2 | | 3 | | 4 | | 5 | | 6 | | Participants with Positive Responses (Options 4–6) | |
|---|---|---|---|---|---|---|---|---|---|---|---|---|---|---|
| | Strongly Disagree | | Disagree | | Slightly Disagree | | Slightly Agree | | Agree | | Strongly Agree | | | |
| | n | % | n | % | n | % | n | % | n | % | n | % | n | % |
| I have a good mastery of the curriculum (n = 1313) | 4 | 0.30 | 22 | 1.68 | 148 | 11.27 | 514 | 39.15 | 573 | 43.64 | 52 | 3.96 | 1139 | 86.75 |
| I prepared well for the lessons (n = 1311) | 1 | 0.08 | 15 | 1.14 | 114 | 8.70 | 488 | 37.22 | 608 | 46.38 | 85 | 6.48 | 1181 | 90.08 |
| My teaching skills were good (n = 1298) | 2 | 0.15 | 19 | 1.46 | 111 | 8.55 | 531 | 40.91 | 571 | 43.99 | 64 | 4.93 | 1166 | 89.83 |
| I have good professional attitudes (n = 1298) | 1 | 0.08 | 6 | 0.46 | 38 | 2.93 | 358 | 27.58 | 791 | 60.94 | 104 | 8.01 | 1253 | 96.53 |
| I was very involved (n = 1297) | 1 | 0.08 | 8 | 0.62 | 70 | 5.40 | 340 | 26.21 | 754 | 58.13 | 124 | 9.56 | 1218 | 93.91 |
| I gained a lot during the course of instruction (n = 1299) | 3 | 0.23 | 35 | 2.69 | 151 | 11.62 | 518 | 39.88 | 522 | 40.18 | 70 | 5.39 | 1110 | 85.45 |
| I cared for the students (n = 1300) | 0 | 0.00 | 4 | 0.31 | 14 | 1.08 | 243 | 18.69 | 831 | 63.92 | 208 | 16.00 | 1282 | 98.62 |
| I was ready to offer help to students when needed (n = 1299) | 0 | 0.00 | 3 | 0.23 | 10 | 0.77 | 163 | 12.55 | 849 | 65.36 | 274 | 21.09 | 1286 | 99.00 |
| I had much interaction with the students (n = 1300) | 0 | 0.00 | 11 | 0.85 | 70 | 5.38 | 445 | 34.23 | 651 | 50.08 | 123 | 9.46 | 1219 | 93.77 |
| Overall speaking, I have very positive evaluation of myself as an instructor (n = 1303) | 0 | 0.00 | 8 | 0.61 | 44 | 3.38 | 366 | 28.09 | 796 | 61.09 | 89 | 6.83 | 1251 | 96.01 |

with that of the second year of implementation. The findings indicate an increase in the perceived helpfulness in all items. Among them, the increase in seven items was statistically significant (Table 4).

Fourth, 90.22% of the respondents would recommend the program to students with similar needs. Fifth, 81.92% of the respondents expressed that they would teach similar courses again in the future. Finally, 82.45% of the respondents indicated that the program had helped their professional development (Table 5).

## DISCUSSION

In the current study, the subjective outcome evaluation findings based on program implementers' perspective showed that a high proportion of the respondents had positive perceptions towards the program and themselves; roughly four-fifths of the implementers regarded the program as helpful to the

**TABLE 3**
**Perceived Effectiveness of the Program by the Implementers**

| The extent to which the Tier 1 Program (i.e., the program in which all students have joined ) has helped your students: | 1 Unhelpful | | 2 Not Very Helpful | | 3 Slightly Helpful | | 4 Helpful | | 5 Very Helpful | | Participants with Positive Responses (Options 3–5) | | Participants with Positive Responses (Options 4–5) | |
|---|---|---|---|---|---|---|---|---|---|---|---|---|---|---|
| | n | % | n | % | n | % | n | % | n | % | n | % | n | % |
| It has strengthened students' bonding with teachers, classmates and their families (n = 1305) | 5 | 0.38 | 131 | 10.04 | 646 | 49.50 | 473 | 36.25 | 50 | 3.83 | 1169 | 89.58 | 523 | 40.08 |
| It has strengthened students' resilience in adverse conditions (n = 1304) | 5 | 0.38 | 185 | 14.19 | 651 | 49.92 | 423 | 32.44 | 40 | 3.07 | 1114 | 85.43 | 463 | 35.51 |
| It has enhanced students' social competence (n = 1305) | 4 | 0.31 | 90 | 6.90 | 529 | 40.54 | 600 | 45.98 | 82 | 6.28 | 1211 | 92.80 | 682 | 52.26 |
| It has improved students' ability in handling and expressing emotions (n = 1306) | 4 | 0.31 | 138 | 10.57 | 566 | 43.34 | 535 | 40.96 | 63 | 4.82 | 1164 | 89.13 | 598 | 45.79 |
| It has enhanced students' cognitive competence (n = 1302) | 6 | 0.46 | 178 | 13.67 | 607 | 46.62 | 450 | 34.56 | 61 | 4.69 | 1118 | 85.87 | 511 | 39.25 |
| Students' ability to resist harmful influences has been improved (n = 1304) | 8 | 0.61 | 203 | 15.57 | 613 | 47.01 | 432 | 33.13 | 48 | 3.68 | 1093 | 83.82 | 480 | 36.81 |
| It has strengthened students' ability to distinguish between the good and the bad (n = 1318) | 4 | 0.30 | 120 | 9.10 | 567 | 43.02 | 558 | 42.34 | 69 | 5.24 | 1194 | 90.59 | 627 | 47.57 |
| It has increased students' competence in making sensible and wise choices (n = 1316) | 4 | 0.30 | 166 | 12.61 | 607 | 46.12 | 489 | 37.16 | 50 | 3.80 | 1146 | 87.08 | 539 | 40.96 |
| It has helped students to have life reflections (n = 1305) | 9 | 0.69 | 217 | 16.63 | 541 | 41.46 | 472 | 36.17 | 66 | 5.06 | 1079 | 82.68 | 538 | 41.23 |
| It has reinforced students' self-confidence (n = 1305) | 11 | 0.84 | 234 | 17.93 | 592 | 45.36 | 412 | 31.57 | 56 | 4.29 | 1060 | 81.23 | 468 | 35.86 |
| It has increased students' self-awareness (n = 1315) | 4 | 0.30 | 95 | 7.22 | 529 | 40.23 | 605 | 46.01 | 82 | 6.24 | 1216 | 92.47 | 687 | 52.24 |
| It has helped students to face the future with a positive attitude (n = 1316) | 16 | 1.22 | 202 | 15.35 | 600 | 45.59 | 457 | 34.73 | 41 | 3.12 | 1098 | 83.43 | 498 | 37.84 |
| It has helped students to cultivate compassion and care about others (n = 1305) | 7 | 0.54 | 191 | 14.64 | 562 | 43.07 | 500 | 38.31 | 45 | 3.45 | 1107 | 84.83 | 545 | 41.76 |
| It has encouraged students to care about the community (n = 1305) | 16 | 1.23 | 265 | 20.31 | 605 | 46.36 | 383 | 29.35 | 36 | 2.76 | 1024 | 78.47 | 419 | 32.11 |
| It has promoted students' sense of responsibility in serving the society (n = 1303) | 14 | 1.07 | 257 | 19.72 | 611 | 46.89 | 382 | 29.32 | 39 | 2.99 | 1032 | 79.20 | 421 | 32.31 |
| It has enriched the overall development of the students (n = 1314) | 5 | 0.38 | 80 | 6.09 | 588 | 44.75 | 572 | 43.53 | 69 | 5.25 | 1229 | 93.53 | 641 | 48.78 |

program participants. The present findings were consistent with the evaluation study in the first year of the Full Implementation Phase[1], and also the subjective outcome evaluation findings based on program participants in the first and second year of the Full Implementation Phase[5,6]. In short, the previously reported findings were replicated in the present study.

**TABLE 4**
**Significant Increase in Positive Responses in Year 2007/08**

| Item | Year 2006/07 | | Year 2007/08 | | Sig. (Two-Tailed) $p \leq 0.05$ |
|---|---|---|---|---|---|
| | Mean | SD | Mean | SD | |
| It has enhanced students' cognitive competence | 0.359 | 0.265 | 0.414 | 0.266 | 0.035 |
| Students' ability to resist harmful influences has been improved | 0.317 | 0.260 | 0.383 | 0.255 | 0.008 |
| It has strengthened students' ability to distinguish between the good and the bad | 0.429 | 0.278 | 0.490 | 0.258 | 0.02 |
| It has helped students to face the future with positive attitude | 0.348 | 0.263 | 0.402 | 0.258 | 0.035 |
| It has helped students to cultivate compassion and care about others | 0.349 | 0.271 | 0.428 | 0.252 | 0.002 |
| It has encouraged students to care about the community | 0.256 | 0.236 | 0.332 | 0.246 | 0.001 |
| It has promoted students' sense of responsibility in serving the society | 0.259 | 0.241 | 0.339 | 0.260 | 0.001 |

**TABLE 5**
**Other Aspects of Subjective Outcome Evaluation Based on the Views of the Respondents**

Recommend the P.A.T.H.S. program to others students with similar needs and condition (n = 1299)

| 1 | | 2 | | 3 | | 4 | | Participants with Positive Responses (Options 3–4) | |
|---|---|---|---|---|---|---|---|---|---|
| Definitely Will Not Suggest | | Will Not Suggest | | Will Suggest | | Definitely Will Suggest | | | |
| n | % | n | % | n | % | n | % | n | % |
| 12 | 0.92 | 115 | 8.85 | 1043 | 80.29 | 129 | 9.93 | 1172 | 90.22 |

Willingness to teach similar programs again in the future (n = 1289)

| 1 | | 2 | | 3 | | 4 | | Participants with Positive Responses (Options 3–4) | |
|---|---|---|---|---|---|---|---|---|---|
| Definitely Will Not Teach | | Will Not Teach | | Will Teach | | Definitely Will Teach | | | |
| n | % | n | % | n | % | n | % | n | % |
| 20 | 1.55 | 213 | 16.52 | 931 | 72.23 | 125 | 9.70 | 1056 | 81.92 |

Helpfulness of the program in facilitating professional growth (n = 1299)

| 1 | | 2 | | 3 | | 4 | | 5 | | Participants with Positive Responses (Options 3–5) | |
|---|---|---|---|---|---|---|---|---|---|---|---|
| Unhelpful | | Not Very Helpful | | Slightly Helpful | | Helpful | | Very Helpful | | | |
| n | % | n | % | n | % | n | % | n | % | n | % |
| 23 | 1.77 | 205 | 15.78 | 583 | 44.88 | 430 | 33.10 | 58 | 4.46 | 1071 | 82.45 |

Furthermore, the findings were also in line with the evaluation findings based on objective outcome evaluation, process evaluation, interim evaluation, and case study on the Full Implementation Phase[7,8,9,10]. In general, the overall picture based on the subjective outcome evaluation findings obtained from different sources was positive. The present findings further demonstrate the respondents' positive evaluation on the implementation of the Tier 1 Program of the Project P.A.T.H.S.

In the second year of implementation, there was an increase in the perceived helpfulness of the program covering all items, and nearly half of them were statistically significant. The increase may have been due to the accumulation of experience from the first year of implementation, which is likely to contribute to a smoother implementation in the second year. There are several possible factors that led to the observed findings. First, the implementers had better mastery of the program rationale and content. Second, the implementers were familiar with the student-centered approach, group facilitation skills, and more experiential approach that involved students' active participation. Their classroom experience consolidated what they had learned during the implementers' training provided by the program and led to a better facilitation. Third, the implementers had a deeper understanding of their students' interests and needs. Fourth, teachers and social workers could better communicate, collaborate, and give mutual support, which in turn shared the workload and alleviated their pressure. Fifth, the schools were more experienced in offering administrative support to the program and the implementers. Finally, the high proportion of positive feedback received in the first year encouraged the schools and the implementers to invest more resources, time, and effort in launching the program, which resulted in a greater and more positive impact on students and the perceived effectiveness on the program.

In line with the findings of Hui's study[2], the present study highlighted the importance of the implementers' role and the schools' support to the program implementation and results. These findings have practical implications. Continuity in the program is important. This does not mean that the schools simply continue to launch the implementation. In order to utilize the human resources, experiences, and resources fully, schools may need to consider the formation of an ongoing team that specializes in carrying out the program in their own schools. The group of teachers and social workers takes charge of the whole program. The accumulation of experience and mutual support within the team would maximize the impact of the program on students, and the efficiency and effectiveness in preparing and carrying out the program. This observation is also in line with the findings of several case studies that "people" (program implementers in particular) is a very important factor that influences the quality of program implementation[11].

Aside from the school system, as aforementioned, based on the perspective of the ecological model, the participation of parents, the community, and society is also essential to teenagers' positive development[12]. It is pertinent to include some supplementary activities or projects in the program to encourage parental involvement. The school can encourage parents to become involved in these activities and strengthen the home-school partnership in cultivating the students' positive development. The future implementation of P.A.T.H.S. may involve media, through disseminating more positive values and promoting positive youth development, culture, and atmosphere. Further collaboration with government agencies with similar vision and mission is also pertinent.

Another point to be addressed is that, when comparing the first and the second year data of the implementers' ratings on which the program was "helpful" or "very helpful" to participants, the ranking was similar, with "enhancing students' social competence", "increasing students' self-awareness", and "enriching the overall development of the students" always in the top three. "Enhancing students' social competence" had the highest rating in both years. A possible contributing factor is that, apart from having the lessons on "Social Competence", students were exposed to the training in this aspect in most of the lessons of the program. Many activities (e.g., group discussion, group activities, role play, etc.) required students to cooperate and collaborate with their classmates, which eventually improved their interpersonal skills and relationships with others. The case of promoting students' self-awareness is similar. Students' "self-awareness" can be enhanced through not only the designated lessons, but also the encouragement on, and opportunities for, self-reflection in the program. The findings revealed that this kind of program design is effective, in the sense that the constructs are inter-related, offering opportunities for students to consolidate and practice what they had learned in one lesson during another lesson. The more the students are exposed to the environment that enables them to consolidate and practice what they have learned, the greater improvement the students will achieve.

However, it was also found that encouraging students' to care about the community and promoting students' sense of responsibility in serving the society earned the lowest ranking in both years. Such findings are expected. Prosocial aspects and the social responsibility of students are generally weaker in Hong Kong students, as there is a trend that teenagers tend to amplify their rights and undermine their social responsibilities. Nevertheless, there was a significant increase in responses on "helpful" and "very helpful" in the second year. These findings also revealed the increased emphasis on service learning in schools and society. The secondary schools see the importance and benefits of participating in volunteering service. Some schools require their students to complete a certain amount of "social service hours" in an academic year as an accomplishment and learning experience. The "Prosocial Norms" and "Prosocial Involvement" advocate in the Program also brings awareness to the schools in order to promote students' prosocial attitude and behavior, as well as social responsibility.

At the same time, the government has been injecting resources to promote civic education. For example, the Commission on Youth is responsible for enhancing the civic awareness of young people and their participation in community affairs[13]. The theme of the Youth Summit in 2004 was "The Voice and Power of the New Generation", which advocated a balanced attention to both rights and responsibilities[14]. Other examples are the Volunteer Movement launched by the Social Welfare Department of HKSAR, with an objective to build a caring community, promoting the positive values of self-fulfillment, maximizing community resources, and enhancing the sense of social belonging[15]; as well as the Agency for Volunteer Service (AVS), which plays a proactive and pivotal role in the promotion and development of volunteerism, and to develop partnership with all sectors of the community to provide value added and quality volunteer service[16].

There are several strengths in the current study. First, the subjective outcome evaluation findings were based on a large sample size (n = 1324). The big sample size enhanced the generalizability of the research findings to other student populations in Hong Kong. Second, the psychometric properties of the measurement were strong. The analysis showed that rating items of the questionnaire were inter-related and reliable, with reference to the sections and the whole scale. Third, different aspects of subjective outcome, including views of the program, program implementers, perceived effectiveness, and overall satisfaction, were covered. Finally, the present findings reinforce the validity of the previous evaluation findings based on different student cohorts[17,18,19,20].

Although the results are promising, it is noteworthy that there are several limitations of the study. This is a self-report study. The respondents may tend to, consciously or unconsciously, respond in a nice manner for the sake of social desirability. However, since the reports were submitted by the participating schools anonymously, the possibility that the implementers reported in an overcooperative manner was not high. Besides, negative ratings were also recorded. Hence, the impact of the limitation of self-report study in the current evaluation may still exist, although should not be great. On the other hand, the program has only launched for 2 years. More years of implementation and longitudinal studies have to be carried out in order to investigate the long-term positive impact of the program. Despite these limitations, the present findings suggest that the Tier 1 Program and its implementation were perceived in a positive manner by the program implementers, and the implementers perceived the program beneficial to the development of the students and themselves.

## ACKNOWLEDGMENTS

The preparation for this paper and the Project P.A.T.H.S. were financially supported by The Hong Kong Jockey Club Charities Trust.
